# PI3-kinase inhibition synergistically promoted the anti-tumor effect of lupeol in hepatocellular carcinoma

**DOI:** 10.1186/1475-2867-13-108

**Published:** 2013-11-01

**Authors:** Fen Liu, Yan He, Yong Liang, Lijun Wen, Yongming Zhu, Yan Wu, Lixiang Zhao, Yunsen Li, Xinliang Mao, Haiyan Liu

**Affiliations:** 1 Laboratory of Cellular and Molecular Tumor Immunology, Jiangsu Key Laboratory of Infection and Immunity, Institutes of Biology and Medical Sciences, Soochow University, Suzhou 215123, P. R. China; 2 Cyrus Tang Hematology Center, Department of Hematology, Jiangsu Institute of Hematology, the First Affiliated Hospital, Soochow University, Suzhou 215006, P. R. China; 3 School of Pharmacy, Medical College, Soochow University, Suzhou 215123, P. R. China; 4 Present address: Oncology Institute, The Fourth People’s Hospital of Wuxi, Wuxi 214062, P. R. China; 5 Clinical Laboratory, Department of Hematology, the Affiliated Huai’an Hospital of Xuzhou Medical College, Huai’an 223002, P. R. China

**Keywords:** Hepatocellular carcinoma, Lupeol, S14161, PI3-kinase/Akt signaling

## Abstract

**Background:**

Lup-20(29)-en-3H-ol (Lupeol), a dietary triterpene, has been shown to possess multiple pharmacological activities including anti-tumor effects

**Methods:**

In the current study, we noted that low doses of lupeol (<40 μM) promoted the growth of hepatocellular carcinoma (HCC) cells with a significant activation of the PI3-kinase/Akt signaling pathway. We further investigated the combined anti-tumor effect of lupeol and S14161, a newly identified PI3-Kinase inhibitor *in vitro* and *in vivo*

**Results:**

The results demonstrated that lupeol and S14161 could exert a synergistic antitumor effect resulting in chemo-sensitization of HCC to low doses of lupeol. Using an *in vivo* HCC model, we further demonstrated that lupeol and S14161 synergistically inhibited tumor growth without any adverse effects on body weight

**Conclusion:**

Our studies showed that the activation of PI3-kinase/Akt pathway resulted in the tumor-promoting effect with low doses of lupeol. Combining PI3-kinase inhibitor with lupeol could synergistically augment the anti-tumor effect of lupeol and might be an applicable strategy for HCC therapy.

## Background

Hepatocellular carcinoma (HCC) becomes the fifth most frequent cancer and the third most common cause of cancer-related mortality in the world, preceded only by lung cancer and stomach cancer [1-4]. Asian countries account for nearly 78% of the roughly 600,000 cases of HCC reported globally each year [5]. Although the prognosis of patients with HCC has marginally improved, a historical 5-year survival rate worldwide is still less than 5%, mainly because of a high potential for vascular invasion, metastasis, and recurrence even after surgical resection [6]. Various approaches have been developed, curative effect is far from perfect due to the highly chemoresistant nature of the tumor and the toxicity of chemotherapeutic agents [4,7-11]. Therefore, efforts are urgently needed to explore more effective therapeutic agents for treating HCC.

There is a growing interest in dietary substances obtained from natural products because of their minimal toxicity. Emphasis has been placed on triterpenes, due to their wide spectrum of biological activities. They could selectively or preferentially eliminate cancer cells by inhibiting cell cycle progression and by causing apoptosis [12]. One such triterpene which has gained wide attention is lupeol [Lup-20(29)-en-3β-ol]. Extensive research over the last three decades has revealed various important pharmacological activities of lupeol under *in vitro* and *in vivo* conditions, including anti-inflammation, anti-arthritis, anti-diabetes, anti-heart diseases, anti-renal toxicity, anti-hepatic toxicity and anti-cancer [13-15]. Lupeol has been reported not only to induce differentiation and inhibit the growth of melanoma and leukemia cells [16-19], but also to inhibit tumor promotion in two-stage mouse skin carcinogenesis through modulating NF-κB and PI3-kinase (PI3K)/Akt pathways [20], and to inhibit growth and induce apoptosis in both prostate [21] and pancreatic cancers [22]. Recent studies have also shown that lupeol induced apoptosis of HCC cells SMMC7721 by down-regulating death receptor 3 (DR3) [23], and also had *in vivo* and *in vitro* therapeutic effect for HCC by targeting liver tumor-initiating cells (T-ICs) through modulating PTEN-Akt-ABCG2 pathway [24]. Our previous work also proved anti-HCC efficacy of lupeol and a combined effect with rTRAIL in inducing chemo-sensitization of HCC [25]. Meanwhile, lupeol exhibited very low toxicity. Lupeol administered orally in a dose of 2 g/kg body weight has been reported to produce no adverse effects in rats and mice [15]. However, the toxicity has not been examined in human. On the other hand, our previous results [25] showed that lupeol could also reduce the cell viability of the normal human liver cells with an IC_50_ of 90 μmol/L, suggesting that lupeol could exert toxic effect on normal cells. Lupeol concentrations of less than 30 μmol/L do not affect the normal liver cell viability. Lupeol has also been shown by numerous studies to have anti-inflammatory activity in rats and mice at the dose of 25-200 mg/kg [26-28]. Therefore, high doses of lupeol could also inhibit anti-tumor immune responses. Therefore, low dose of lupeol is desirable because it can minimize the toxicity to normal cells and the immune suppressive effect of lupeol if the anti-tumor effect could also be achieved. In the current study, we found that low doses of lupeol could promote tumor growth *in vitro* and had a very minimal effect on HCC *in vivo.* We further exploited the underlying mechanisms and demonstrated a synergistic effect of combination treatment with low doses of lupeol and PI3K inhibitor in HCC, which made low dose lupeol possible for tumor treatment.

PI3K/Akt pathway plays an important role in various types of cancers, including HCC. Akt is important in protecting the cells from various types of apoptotic stimuli and regulating cell proliferation and cell cycle by interacting, either directly or indirectly, with numerous other regulatory proteins [29,30]. Blockage of Akt signaling by some reagents results in programmed cell death and growth inhibition of tumor cells [31-34]. Therefore, targeted therapies against specific components of this pathway are expected to be efficacious as single agents or in combination in a variety of human cancers. Up to now, many inhibitors of PI3K/Akt pathway have been developed. LY294002 and wortmannin both target the catalytic site p110 of PI3K. Due to their unfavorable pharmaceutical properties, toxicity, and crossover inhibition of other lipid and protein kinases, they were not extensively used in clinical trials [35]. Recently, 8-ethoxy-2-(4-fluorophenyl)3-nitro-2H-chromene (S14161) showed potent anti-leukemia and anti-myeloma activity *in vitro* and inhibited *in vivo* tumor growth [36]. S14161 has been shown to have no effect on the cell viability of the normal hematopoietic cells with the concentration as high as 25 μmol/L and no effect on body weight with 100 mg/kg/day intraperitoneal injection for 10 days. The effect of S14161 on HCC has not been determined.

In the present study, we unexpectedly discovered that low doses of lupeol promoted cell growth of HCC cells through the activation of PI3K/Akt pathway. To further improve the anti-tumor efficacy of lupeol, we combined lupeol treatment with S14161. The results demonstrated that lupeol and S14161 could exert synergistic effects inhibiting tumor growth *in vitro* and *in vivo*. Our results provided evidence that PI3-kinase/Akt signaling pathway activation promoted tumor growth by low doses of lupeol. Combining PI3K inhibition and lupeol treatment could provide safer and more effective anti-tumor therapeutic regimen.

## Methods

### Cell lines and culture

Human HCC cell lines, HepG2 and SMMC7721, were purchased from Cell Bank, Chinese Academy of Sciences (Shanghai, China). They were maintained in Dulbecco’s modified Eagle’s medium (DMEM) with high glucose (Gibco BRL, Grand Island, NY) supplemented with 10% heat-inactivated fetal bovine serum (Gibco BRL, Grand Island, NY), 10mg/ml penicillin G and 50 μg/ml treptomycin (Shanghai Sangon Biological Engineering Technology & Services Co., Shanghai, China) at 37°C in a humidified atmosphere containing 5% CO_2_. Cells were harvested using 0.25% trypsin/EDTA (Invitrogen, Carlsbad, CA).

### Antibodies and reagents

Lupeol was purchased from Sigma-Aldrich (St. Louis, MO) and a stock solution of lupeol (30 mmol/L) was prepared by resuspension in warm alcohol and dilution in DMSO at 1:1 ratio.

Antibodies against β-actin was purchased from BD Pharmingen (Franklin Lakes, NJ). Antibodies against PI3/K p110δ, phospho-Akt (Thr308) and total-Akt were purchased from Cell Signal Technology (Boston, MA). Cytoplasmic Protein Extraction Kit and BCA Protein Assay Kit were purchased from Beyotime (Nantong, China).

### Cell viability assay

The effect of Lupeol and/or S14161 on cell viability was determined by 3-(4,5-dimethylthiazol-2-yl)-2,5-diphenyltetrazoliumbromide (MTT) assay. Cells were plated at 3 × 10^3^ per well in 100 μl of complete culture medium in 96-well cell culture plates 24 h before the assay. Then cells were treated with different concentrations of related compounds for 48 h. Each concentration was repeated in 5 wells. After incubation for 48 h, 20 μl MTT (5 mg/ml in PBS) was added to each well and incubated for 4 h; then the medium was removed, 0.1 mL of buffered DMSO was added to each well. The absorbance was recorded on a microplate reader at the wavelength of 490 nm. The effect on cell growth inhibition was assessed as percent cell proliferation inhibition wherein vehicle-treated cells were taken as 0% inhibition.

### Protein preparation and western blot analysis

HCC cells (50% confluent) were treated with 10, 20, 30 μmol/L of lupeol, 1 μmol/L, 3 μmol/L of S14161 alone or in combination with 20 μmol/L lupeol for 48 h in 10% fetal bovine serum-DMEM. Cells were then harvested and cell lysates were prepared using Cytoplasmic Protein Extraction Kit (Beyotime, Nantong, China) and stored at -80°C for later use. The protein content in the lysates was measured by BCA Protein Assay Kit (Beyotime, Nantong, China). For Western blot analysis, 25 μg of protein were resolved over 12% tris-glycine polyacrylamide gels under nonreduced conditions, transferred onto PVDF membranes, and subsequently incubated in blocking buffer (5% nonfat dry milk in PBS) overnight at 4°C. The blots were incubated with appropriate primary antibody, washed, and incubated with horseradish peroxidase (HRP)-conjugated secondary antibody (Dako, Carpinteria, CA). The blots were detected with chemiluminescence (ECL-Kit, Beyotime, Nantong, China) followed by autoradiography. Relative amounts of proteins were quantified by absorbance analysis. The level was normalized to β-actin, a domestic loading control.

### Animal studies

A total of 2 × 10^6^ SMMC7721 cells suspended in 200 μl PBS were inoculated s.c. into the right flank of 6- to 8-week-old female athymic nude mice (BALB/c-nu/nu). Treatment was started once the size of the xenograft reached approximately 4 × 4 mm. The mice were randomly assigned into four groups, each consisting of 6 mice. They were treated with intraperitoneal injection for 3 weeks of either (1) 20 mg/kg lupeol in 0.1 mL of corn oil, (2) 20 mg/kg S14161 in 0.1 mL corn oil, (3) 20 mg/kg lupeol plus 20 mg/kg S14161 in 0.1 mL corn oil, or (4) 0.1 mL of corn oil alone as the control group. Lupeol was injected three times/week (total 9 times), while S14161 was injected once/day for five continuous days/week (total 15 times). Animals in all the groups were observed for any apparent signs of toxicity, such as weight loss or mortality during the entire period of study. Tumor growth was assessed weekly by measuring the two greatest perpendicular tumor dimensions. Tumor volume was calculated by the formula: tumor volume (mm3) = [tumor length (mm) × tumor width (mm)2]/2 (34). All animals were sacrificed at the end of 5 weeks. Animal studies were carried out in accordance with the national guidelines for animal experiments and were specifically approved by the Ethical Committee of Soochow University. The body weight and the tumor size were carefully monitored and all efforts were made to minimize suffering.

### Statistical analysis

All data represents at least three independent experiments and results were shown as mean ± SD. Statistical differences between two groups were determined by Student’s *t*-test. Analysis of variance (ANOVA) analysis was applied for multiple group comparison. A significant difference was considered as *p* < 0.05.

## Results

### Low doses of lupeol promoted the viability and activated the PI3K/Akt pathway in HCC cell lines

**Figure 1:**
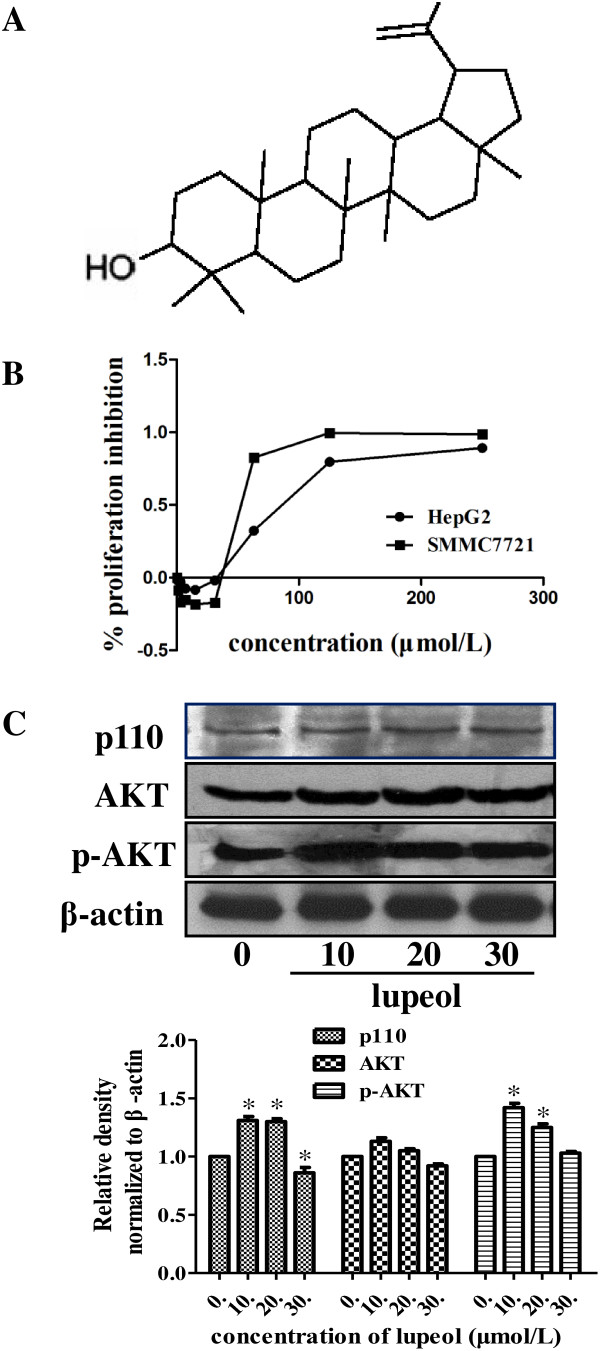
**Low doses of lupeol promoted the viability and activated the PI3K/Akt pathway in HCC cell lines. (A)** The chemical structure of lupeol. **(B)** MTT assay was performed to measure cell viability of HepG2 and SMMC7721 cells at 48 h after lupeol treatment. **(C)** PI3K subunit p110 protein expression and the phosphorylated level of Akt was measured by western blot in SMMC7721 cells treated with low doses of lupeol at doses of 10, 20, 30 μmol/L for 48 h. Equal loading was confirmed by stripping immunoblots and re-probing for β-actin. The quantification results were also shown and the statistical analysis was performed. Data shown were representatives of three independent experiments. * *p* < 0.05.

We and others have previously reported that lupoel could inhibit cell growth of HCC cells in a dose dependent manner [23,25]. Meanwhile, we have also noted that low concentrations of lupeol promoted the viability of HCC cells (Figure 1B). Studies have shown that PI3K/Akt pathway plays an important role in chemical-resistance of various cancers. Western blotting revealed that the protein levels of PI3K p110 and the total and phosphorylated level of Akt were increased with low dose lupeol treatment, especially at 10 and 20 μmol/L (Figure 1C). These data suggested that low doses of lupeol could activate PI3K/Akt pathway, which might be the reason for its promoting effect on HCC cell viability.

### Synergistic anti-HCC effect of S14161 and lupeol *in vitro*

**Figure 2:**
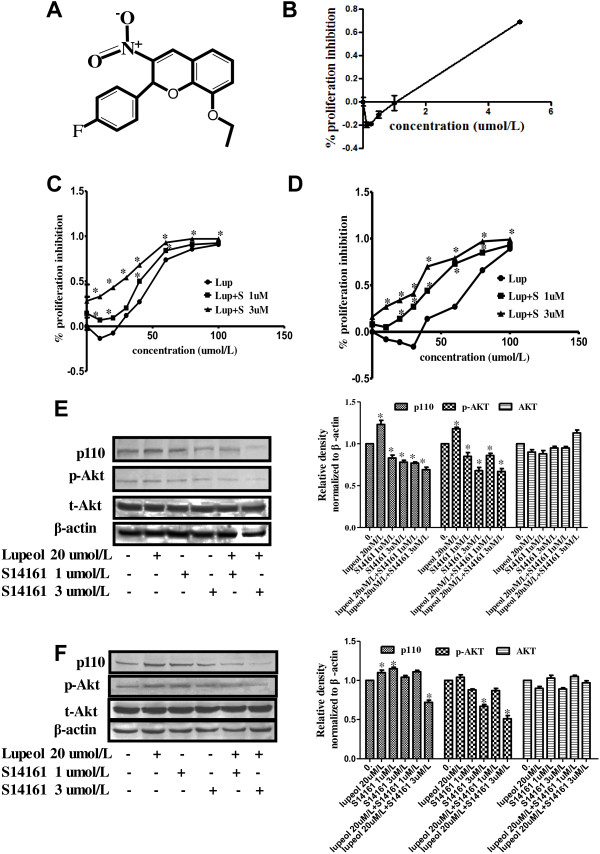
**Synergistic anti-HCC effect of S14161 and lupeol *in vitro*. (A)** The chemical structure of S14161. **(B)** MTT assay was performed to measure cell growth inhibition rate of SMMC7721 at 48 h after S14161 treatment. MTT assay was performed to measure cell growth inhibition rate of **(C)** SMMC7721 cells and **(D)** HepG2 cells 48 h after treated with lupeol alone or in combination with 1 or 3 μmol/L S14161. Expression level of PI3-kinase subunit p110 and the phosphorylated level of Akt in **(E)** SMMC7721 cells and **(F)** HepG2 cells were measured by western blot after single or combination treatment with low dose of lupeol (20 μmol/L) and S14161 (1 μmol/L or 3 μmol/L) for 48 h. Equal loading was confirmed by stripping immunoblots and re-probing for β-actin. Statistical analysis was performed. Results shown were the representatives of three independent experiments. * *p* < 0.05.

To sensitize HCC cells to low doses of lupeol therapy, we evaluated the effect of combining PI3K inhibitor and lupeol treatment. S14161 (Figure 2A) is a newly reported PI3K inhibitor [36], and its chemical structure is similar to that of LY294002, a well-known PI3K inhibitor. Based on the dose–response curves, the IC_50_ of S14161 was calculated as 4 μmol/L for SMMC7721 (Figure 2B). The concentration of 1 μmol/L (no inhibition) and 3 μmol/L (30% inhibition) were used in the following experiments. To examine the effect of combined lupeol and S14161 treatment on HCC cells, SMMC7721 cells were treated by lupeol with doses ranging from 10 to 100 μmol/L at the presence of 1 or 3 μmol/L S14161 (Figure 2C). Interestingly, S14161 at 1 and 3 μmol/L enhanced the cell growth inhibition in SMMC7721 cells treated by lupeol. The IC_50_ was significantly reduced when the cells were treated with both lupeol and S14161 (lupeol alone: 44.9 μmol/L, lupeol + 1 μmol/L S14161: 40.1 μmol/L, lupeol + 3 μmol/L S14161: 27.9 μmol/L). A synergistic effect on HCC cell growth inhibition was observed with the combination treatment, especially with combined low dose lupeol and S14161 (Figure 2C). Similar results were also observed with HepG2 cells (Figure 2D).

We then investigated the activity of the PI3K/Akt pathway with single or combined treatment of low dose lupeol and S14161. As shown in Figure 2E, the expression levels of PI3K subunit p110 and phosphorylated Akt were increased with the 20 μmol/L lupeol treatment. Not surprisingly, the PI3K inhibitor, S14161 slightly reduced the level of phosphorylated Akt at 1 and 3 μmol/L concentrations and this reduction was maintained when S14161 was combined with lupeol treatment. The phosphorylated Akt was also significantly reduced with 3 μmol/L S14161 and the combined treatment with lupeol in HepG2 cells (Figure 2F). These results suggested that PI3K/Akt pathway activation by low doses of lupeol could be reversed by combinational treatment with PI3K inhibitor, S14161.

### Synergistic anti-HCC effect of S14161 and lupeol *in vivo*

**Figure 3:**
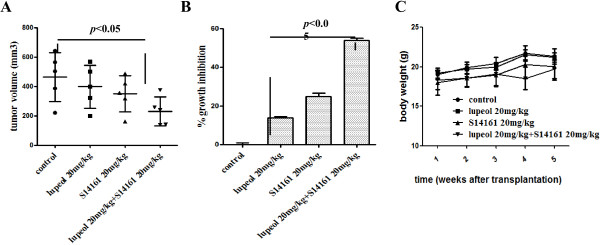
**Synergistic anti-HCC effect of S14161 and lupeol *in vivo*. (A)** Tumor volumes after single or combination treatment with 20 mg/kg lupeol and 20 mg/kg S14161. SMMC7721 xenograft nude mice were treated with lupeol and/or S14161 for 3 weeks, and then were observed for one more week. Tumors were measured and tumor volumes were calculated. **(B)** The growth inhibition rates of tumors after single or combination treatment with 20 mg/kg lupeol and 20 mg/kg S14161. **(C)** Body weights of mice in each group were measured every week for 5 weeks during the treatment. Values represent means ± SD of n = 6. Results shown were the representatives of three independent experiments.

A nude mouse model of HCC was used to assess the *in vivo* anti-tumor effect of S14161 and lupeol. Lupeol at a dose of 20 mg/kg three times per week and S14161 at a dose of 20 mg/kg five times per week were administered to the mice bearing established SMMC7721 tumors for 3 weeks (Figure 3). At the end of the therapy, single therapy with lupeol (399 ± 146 mm^3^) or S14161 (350 ± 123 mm^3^) showed decreased tumor volumes by 14% and 25% compared to the controls (464 ± 160 mm^3^), respectively (Figure 3A&B). In addition, the combination treatment seemed to be more effective than the single treatments. The tumor volume was reduced by 54% (230 ± 98 mm^3^) compared to the controls. Therefore, the combination treatment of S14161 and lupeol synergistically promoted the anti-tumor effects of either treatment alone. To examine the side effects of the combination therapy, the body weights were recorded each weak, and no significant differences in body weights were detected among each treatment groups (Figure 3C). The results demonstrated that combining S14161 and lupeol treatment could synergistically inhibit the HCC tumor growth *in vivo* with little toxicity.

## Discussion and conclusion

Previous studies have focused on the anti-tumor effects and mechanisms of lupeol in HCC. Studies have shown that lupeol induced apoptosis of SMMC7721 cells by down-regulating death receptor 3 (DR3) [23]. Lupoel could also target liver tumor-initiating cells (T-ICs) though modulating PTEN-Akt-ABCG2 pathway [24]. Our previous work also proved anti-HCC efficacy of lupeol and a combined effect with rTRAIL in inducing chemo-sensitization of HCC [25]. In this report, we first described the tumor-promoting role of lupeol at low doses. We discovered that PI3K/Akt pathway was activated by low concentrations of lupeol treatment. We further demonstrated that inhibition of the PI3K/Akt pathway enhanced the antitumor effect of lupeol and the combination therapy of lupeol and S14161 synergistically promoted therapeutic effect on HCC.

PI3K/Akt pathway is critically involved in the control of cell growth, cell survival and malignant transformation [37]. Blockage of PI3K/Akt signaling pathway results in programmed cell death and growth inhibition of tumor cells. An Akt inhibitor, perifosine, showed synergistic antitumor effect with cisplatin in HepG2 cells through down-regulating the expression of Bcl-2 and up-regulating the level of Bax [34]. A PI3K inhibitor, LY294002, also showed synergistic antitumor effect with cisplatin in human pancreatic cancer cells by down-regulating the phosphorylated levels of Bad protein [38]. Recently, S14161 showed potent anti-leukemia and anti-myeloma activity *in vitro* and inhibited *in vivo* tumor growth through inhibiting the activity of PI3K [36]. Lupeol has also been reported to inhibit skin cancer in CD-1 mice through inhibition of TPA-induced activation of PI3K and phosphorylated level of Akt at Thr308 [20]. However, this study was performed *in vivo* at relatively high concentrations of lupeol (1 or 2 mg/mouse). We have also observed inhibition of Akt phosphorylation at 50 μmol/L lupeol or higher *in vitro* (data not shown). On the other hand, low doses of lupeol could promote PI3K/Akt pathway, especially at 10-20 μmol/L concentrations, which suggested that lupeol could function through different targets that had opposite effects on PI3K/Akt pathway with different affinities.

Many natural products have been found to have multiple targets, which allow them to have multiple pharmacological activities. Lupeol has been shown to exhibit anti-inflammatory, anti-microbial, anti-protozoal, anti-tumor, anti-angiogenic and cholesterol lowering activities [39]. The mechanism of the anti-tumor effect of lupeol was initially thought to be inhibiting NFκB [22]. Wnt/β-catenin pathway was also found to be suppressed by lupeol in treating human melanoma cells [19]. Lupoel could also target liver tumor-initiating cells (T-ICs) though modulating PTEN-Akt-ABCG2 pathway [24]. Recently, lupeol has been found to be a novel androgen receptor inhibitor that may be effective in treating prostate cancer [40]. Therefore, many signaling pathways may work together to exert the anti-tumor effect of lupeol. We propose based on our findings that lupeol may have a target with high affinity that promotes PI3K/Akt activities and tumor cell growth at low doses. At high concentrations of lupeol, the low affinity targets of lupeol dominate and regulate the signaling pathways that eventually lead to the suppression of tumor cell growth.

Taken together, our results demonstrated that lupeol could target to activate PI3-kinase/Akt pathway and promote tumor cell growth at low doses. Combination therapy of lupeol and a PI3-kinase inhibitor, S14161, could synergistically enhance the antitumor effect of lupeol *in vitro* and *in vivo*. Therefore, our results support the notion that lupeol combining with PI3-kinase inhibitor may provide more effective anti-HCC regimen.

## Competing interests

The authors declare no conflicts of interest.

## Authors’ contributions

FL carried out the cellular and animal studies and drafted the manuscript. YH, YL and LW participated in the animal studies. YZ participated in the design of the study. YW and LZ participated in the western blot analysis. YL and XM provided the critical material and participated in the design of the study. HL conceived of the study, and participated in its design and coordination and helped to draft the manuscript. All authors read and approved the final manuscript.
